# Evaluation of MiR-1908-3p as a novel serum biomarker for breast cancer and analysis its oncogenic function and target genes

**DOI:** 10.1186/s12885-020-07125-4

**Published:** 2020-07-10

**Authors:** Youzhi Zhu, Qingshui Wang, Yun Xia, Xiaoxue Xiong, Shuyun Weng, Huizhen Ni, Yan Ye, Ling Chen, Junyu Lin, Yajuan Chen, Haitao Niu, Xiangjin Chen, Yao Lin

**Affiliations:** 1 Department of Thyroid and Breast Surgery, The First Affiliated Hospital of Fujian Medical University, Fuzhou, China; 2 Key Laboratory of OptoElectronic Science and Technology for Medicine of Ministry of Education, College of Life Sciences, Fujian Normal University, Fuzhou, China; 3 The Engineering Technology Research Center of Characteristic Medicinal Plants of Fujian, Ningde Normal University, Ningde, China

**Keywords:** Breast cancer, miR-1908-3p, Proliferation, Migration, Invasion

## Abstract

**Background:**

Breast cancer is one of the most common tumors for women globally. Various miRNAs have been reported to play a crucial role in breast cancer, however the clinical significance of miR-1908-3p in breast cancer remains unclear. The present study aimed to explore the role of miR-1908-3p in breast cancer.

**Methods:**

The expression of miR-1908-3p was detected in 50 pairs of breast cancer tissues and adjacent normal tissues, 60 breast cancer patient serum and 60 healthy volunteer serum. The functional roles of miR-1908-3p in breast cancer cells such as proliferation, migration and invasion were evaluated using CCK8, SRB, wound healing and transwell chambers. In addition, bioinformatics tools were used to identify potential targets of miR-1908-3p.

**Results:**

The results showed that the expression of miR-1908-3p were increased in breast cancer tissues and serum compared with normal breast tissues and serum of healthy volunteers respectively. Furthermore, the young breast cancer patients and HER2-positive patients had a higher level of tissues’ miR-1908-3p than elder breast cancer patients and HER2-negative patients, respectively. The young breast cancer patients had a higher level of serum miR-1908-3p than elder breast cancer patients, ROC analysis suggested that miR-1908-3p had the potential as a promising serum diagnostic biomarker of breast cancer. Up-regulation of miR-1908-3p promoted the cells proliferation, migration and invasion while knockdown of miR-1908-3p inhibited these processes in breast cancer cell MCF-7 and MDA-MB-231. The potential target genes of miR-1908-3p in breast cancer included ID4, LTBP4, GPM6B, RGMA, EFCAB1, ALX4, OSR1 and PPARA. Higher expression of these eight genes correlated with a better prognosis for breast cancer patients.

**Conclusions:**

These results suggest that miR-1908-3p may exert its oncogenic functions via suppression of these eight genes in breast cancer.

## Background

Breast cancer is both the most commonly diagnosed cancer and the commonest cause of cancer death among women, which accounts for 630,000 deaths worldwide in 2018 [1]. Despite advances in early detection and development of new therapeutic targets. Although the survival rate of patients with breast cancer has improved, the five-year recurrence rate and five-year survival rate for breast cancer patients with metastases remain high. Therefore, the discovery of new molecular participants in the progress of breast cancer is essential to improve the diagnosis and treatment of breast cancer.

Previous research indicated that numerous miRNAs are involved in the progress of breast cancer [2–7]. MiRNAs are a type of non-coding RNAs containing 21–25 nucleotides, and function as gene regulators by binding to target genes and inhibiting translation [8]. Many of these target genes are involved in fundamental biological processes such as cells differentiation, cells proliferation and cells migration [9–12]. The dysregulated miRNAs play a key role in tumorigenesis and tumor development and are related to poor prognosis in various carcinomas [13–15]. Previous studies have revealed that miR-1908 is an oncogene in glioblastoma [16]. In addition, miR-1908 is also associated with the prognosis of various tumors, such as osteosarcoma [17], hepatoma [18] and glioma [19]. However, the expression, functional roles and target genes of miR-1908-3p in breast cancer progression has not yet been studied.

In this work, miR-1908-3p expression was examined in tissues, TCGA database and the serum of breast cancer patients. The functional roles of miR-1908-3p were also studied. In addition, screening and enrichment analysis of miR-1908-3p target genes were performed to analyze the potential regulatory mechanisms of miR-1908-3p function.

## Methods

### Collection of clinical tissues and serum

The research was composed of 50 breast cancer fresh tissue samples (range from 28 to 83 years) and 50 adjacent breast normal tissue samples who underwent surgical resections at the first affiliated hospital of Fujian medical university between April 2018 and June 2019. The specimens of this study were diagnosed as breast cancer tissues by pathological diagnosis. The extracted samples were immediately placed in liquid nitrogen and then stored at − 80 °C. A total of 60 breast cancer patient serum samples (range from 26 to 81 years) and 60 healthy control serum samples were collected from The First Affiliated Hospital of Fujian Medical University. This study was performed with the approval of the Ethics Committee of the first affiliated hospital of Fujian Medical University and complied with the Helsinki Declaration. All patients and healthy volunteers have signed written informed consent.

### Cell culture and transfection

MCF-10A (normal epithelial cell line), MCF-7(Human breast cancer cell line) and MDA-MB-231(Human breast cancer cell line) were obtained from the ATCC (Manassas, VA, USA). All cells were cultured in Dulbecco’s modified Eagle’s medium containing 10% fetal bovine serum and then incubated at 37 °C in a 5% CO2 environment. These cells underwent mycoplasma testing and STR analyses. Lipofectamine 2000 (Invitrogen, Carlsbad, CA, USA) was used for miRNA transfection experiments [20]. Human breast cancer cell lines MCF-7 and MDA-MB-231 were divided into three groups: control (NC), miR-1908-3p-mimic, miR-1908-3p-inhibitor. miR-1908-3p-mimic and miR-1908-3p-inhibitor were synthesized by GenePharma company (Shanghai, China, Cat. No. B03001 & B01001).

### RNA extraction and real-time polymerase chain reaction

Trizol reagent (Invitrogen, Carlsbad, CA, USA) was used for RNA extraction experiments. The TaqMan MicroRNA Reverse Transcription Kit (Takara, Otsu, Japan) was then used to synthesize cDNA [21]. Real-Time PCR was performed on Applied Biosystems StepOne Plus Real-Time PCR System (Takara, Otsu, Japan) with the PowerUp SYBR Master Mix kit (Thermo Fisher, Shanghai, China,), and the following cycling conditions: 95 °C 10 min, 40 cycles of 95 °C, 30s; 57 °C, 5 s; 72 °C, 15 s. In the detection of miRNA in serum samples, a synthetic elegans nematode miRNA (cel-miRNA-39) was used as an internal control due to the lack of universal endogenous controls. Detailed primer sequence information is listed in Additional file 1.

### Cell proliferation assay

MCF-7 and MDA-MB-231 cells were cultured in a culture plate at a density of 5000 cells per well. After 24 h of incubation, compounds of various concentrations were added to the cell culture medium, and cultured for another 72 h. The CCK8 or SRB assay is used to determine cell proliferation [22].

### Transwell invasion chamber experiment and cell migration experiment

The scratch assay was used to detect the migration of MDA-MB-231 cells and MCF-7 cells. In this experiment, when the degree of cell fusion after transfection was 80%, a 100 μl pipette tip was used for scraping. After washing with PBS, the wound closure was observed, and the cells migration rate was calculated by the ratio of the surface area of the migrated cells to the total surface area.

The Transwell chamber was used to detect the invasion of MDA-MB-231 cells and MCF-7 cells. In this experiment, cells were cultured in an insertion chamber (Corning) with a Matrigel-coated membrane. After the cells were fixed and stained with 0.1% crystal violet, five random fields of each group were selected under the microscope and counted.

### Bioinformatics analysis

TargetScan website (http://www.Targetscan.org/) was used to predict potential target genes for miR-1908-3p [23–26]. The expression of potential target genes for miR-1908-3p in breast cancer were obtained from GEPIA website (http://gepia.cancer-pku.cn/index.html) [27]. The prognosis of miR-1908-3p and potential target genes for miR-1908-3p were obtained from Kaplan-Meier Plotter website (http://kmplot.com/) [28].

### Statistical analysis

Data were analyzed using Prism 5.0 software (Graphpad Software, Inc., La Jolla, CA, USA). Quantification of miR-1908-3p level in breast cancer tissues and serum were calculated with the 2^-∆Ct^ method. Relative quantification of miR-1908-3p expression in breast cancer cell lines was calculated with the 2^-∆∆Ct^ method. The difference of the two lines was calculated using the Grouped analyses (Two-way ANOVA). Receiver operating characteristics (ROC) curve analysis was used to analyze the ability of miR-1908-3p as a serum biomarker for breast cancer patients. A *p* value of < 0.05 indicates statistical significance.

## Results

### Upregulation of miR-1908-3p in breast cancer

**Fig. 1:**
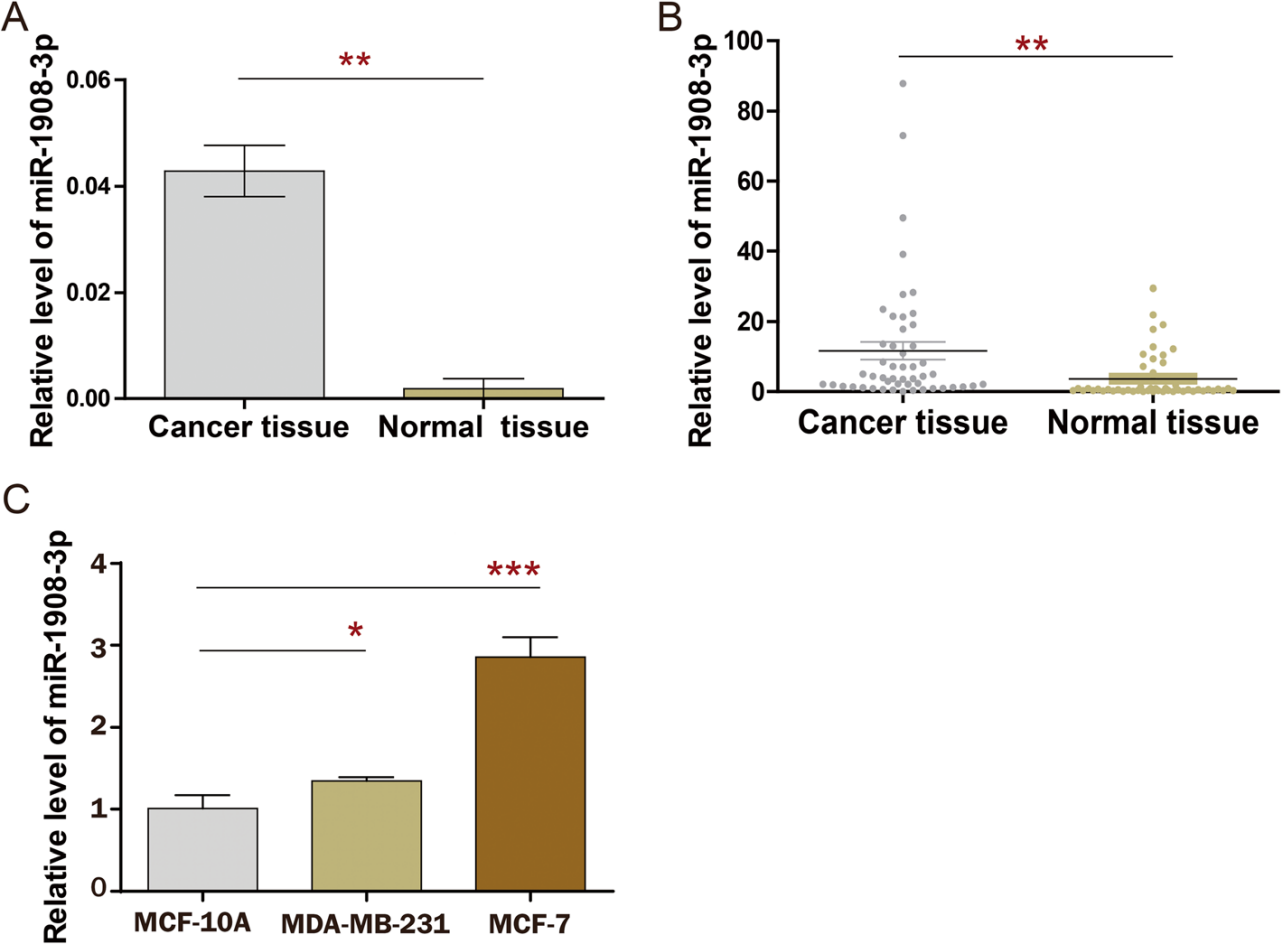
MiR-1908-3p is highly expressed in breast cancer tissues and breast cancer cells. **a** The miR-1908-3p expression level in breast cancer tissues and adjacent normal breast tissues were compared using TCGA. **b** The miR-1908-3p expression level in 50 pairs of fresh breast cancer tissue and adjacent normal tissue was determined by RT-qPCR. Quantification of miR-1908-3p expression were calculated with the 2-∆Ct method. **c** The expression of miR-1908-3p in two breast cancer cell lines and the non-transformed mammary epithelial MCF-10A was determined by RT-qPCR. Relative quantification of miR-1908-3p expression were calculated with the 2-∆∆Ct method. *, *p* < 0.05; **, *p* < 0.01; ***, *p* < 0.001

**Fig. 2:**
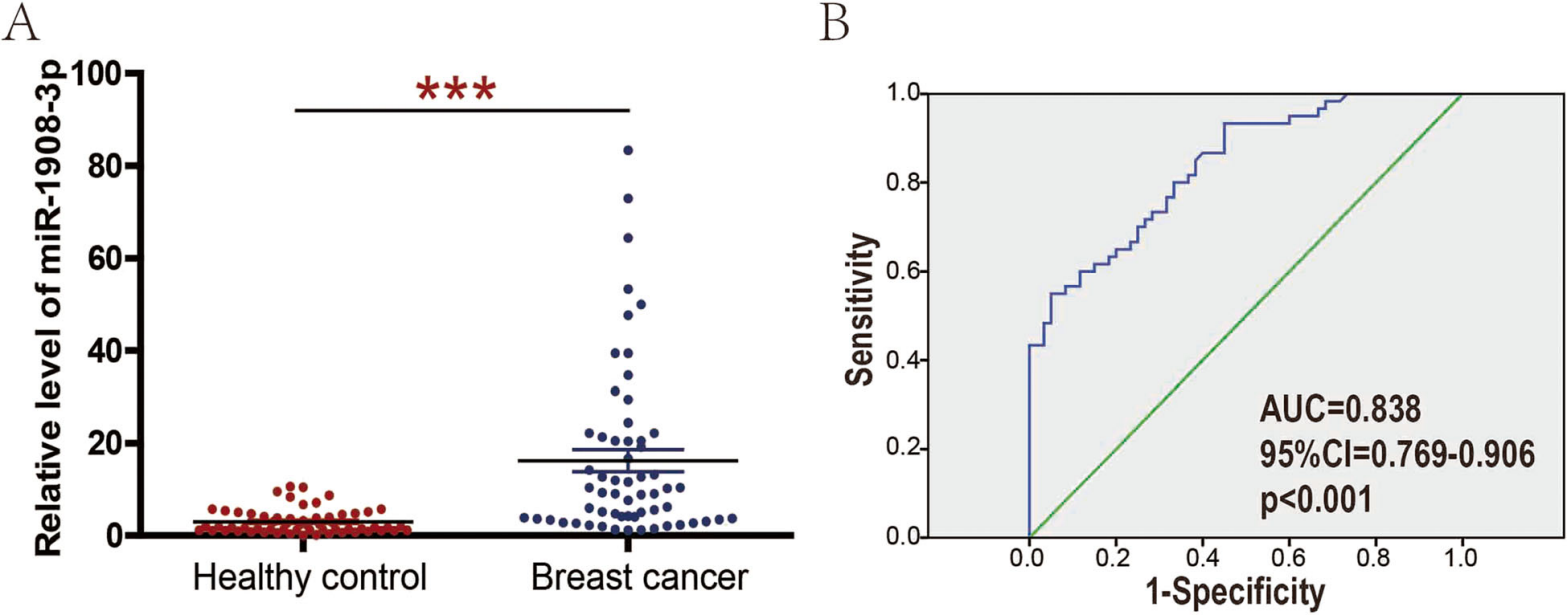
Serum miR-1908-3p level is increased in breast cancer patients compared with healthy donors. **a** The serum miR-1908-3p level in 60 breast cancer patients and 60 healthy donors were determined by RT-qPCR. Quantification of miR-1908-3p expression were calculated with the 2-∆Ct method. **b** High levels of serum miR-1908-3p as a diagnostic marker in patients with breast cancer based on 60 breast cancer patients and 60 healthy donors.***, *p* < 0.001

In order to explore the expression pattern of miR-1908-3p in breast cancer, TCGA dataset was selected for initial screening. Analysis using TCGA data showed that the level of miR-1908-3p was significantly higher in breast cancer tissues than that in normal breast tissues (Fig. 1a, *p* < 0.01). Additionally, detection in the 50 breast cancer tissues samples and 50 matched adjacent normal breast tissues samples further confirmed that miR-1908-3p levels were increased in breast cancer (Fig. 1b, *p* < 0.01). Compared to the normal breast cancer cell line MCF-10A, miR-1908-3p levels were also found to be enhanced in two breast cancer cell lines MCF-7 and MDA-MB-231 (Fig. 1c). The correlations between patients’ clinical characteristics and the levels of miR-1989-3p in tissues are summarized in Table 1. Only age and her-2 status were significantly associated with miR-1908-3p expression in cancer tissues. The breast cancer patients with Age ≤ 40 or positive her-2 status have higher miR-1908-3p levels. Moreover, the expression of miR-1908-3p was significantly higher in the serum of 60 breast cancer patients compared to 60 healthy donors (Fig. 2a). The area under the curve (AUC) of the serum miR-1908-3p was 0.838 (Fig. 2b), suggesting serum miR-1908-3p expression might be a new serum biomarker for breast cancer identification. In addition, the correlations between patients’ clinical characteristics and serum levels of miR-1989-3p are summarized in Table 2. Only age was significantly associated with the miR-1908-3p levels in serum. The breast cancer patients with Age ≤ 40 have higher serum level of miR-1908-3p.

**Table 1:** Clinicopathological variables and the expression of miR-1908-3p in the breast cancer tissues

Variable	Number	Mean of miR-1908-3p expression	*p* value
**Age**			< 0.05
≤ 40	25 (50%)	14.89	
> 40	25 (50%)	5.54	
**metastasis**			0.25
Negative	21 (42%)	6.83	
Positive	29 (58%)	12.66	
**Grade**			0.54
1	3 (6%)	12.49	
2	21 (42%)	6.97	
3	26 (52%)	12.57	
**TNM**			0.66
I/II	36 (72%)	9.53	
III/IV	14 (28%)	11.95	
**Tumor size**			0.84
≤ 2	20 (40%)	8.39	
> 2,≤5	28 (56%)	11.33	
> 5	2 (4%)	12.68	
**ER statue**			0.41
Negative	16 (32%)	7.24	
Positive	34 (68%)	11.61	
**PR statue**			0.3
Negative	19 (38%)	6.36	
Positive	31 (62%)	12.22	
**HER-2**			< 0.01
Negative	35 (70%)	5.59	
Positive	15 (30%)	20.98	
**Molecular Subtype**			0.65
luminal	34 (68%)	11.61	
her-2+	8 (16%)	9.14	
Triple-negative	8 (16%)	5.34	

*Abbreviations*: *ER* estrogen receptor, *PR* progesterone receptor, *HER-2* human epidermal growth factor receptor 2

**Table 2:** Clinicopathological variables and the expression of miR-1908-3p in the serum of breast cancer

Variable	Number	Mean of miR-1908-3p expression	*p* value
**Age**			< 0.001
≤ 40	27 (45%)	30.41	
> 40	33 (55%)	5.18	
**Metastasis**			0.723
Negative	25 (42%)	15.42	
Positive	35 (58%)	17.33	
**Grade**			0.086
1	11 (18%)	15.77	
2	20 (33%)	9.01	
3	29 (49%)	22.00	
**TNM**			0.072
I/II	46 (77%)	16.00	
III/IV	14 (23%)	18.26	
**Tumor size**			0.721
≤ 2	22 (37%)	15.46	
> 2,≤5	35 (58%)	16.42	
> 5	3 (5%)	25.73	
**ER statue**			0.65
Negative	19 (32%)	18.29	
Positive	41 (68%)	15.72	
**PR statue**			0.418
Negative	22 (37%)	19.36	
Positive	38 (63%)	14.89	
**HER-2**			0.489
Negative	52 (87%)	15.81	
Positive	8 (13%)	21.22	
**Molecular Subtype**			0.786
luminal	41 (68%)	15.72	
her-2+	5 (8%)	14.86	
triple-negative	14 (24%)	19.79	

*Abbreviations*: *ER* estrogen receptor, *PR* progesterone receptor, *HER-2* human epidermal growth factor receptor 2

### miR-1908-3p promoted the proliferation, migration, and invasion of breast cancer MCF-7 cells

**Fig. 3:**
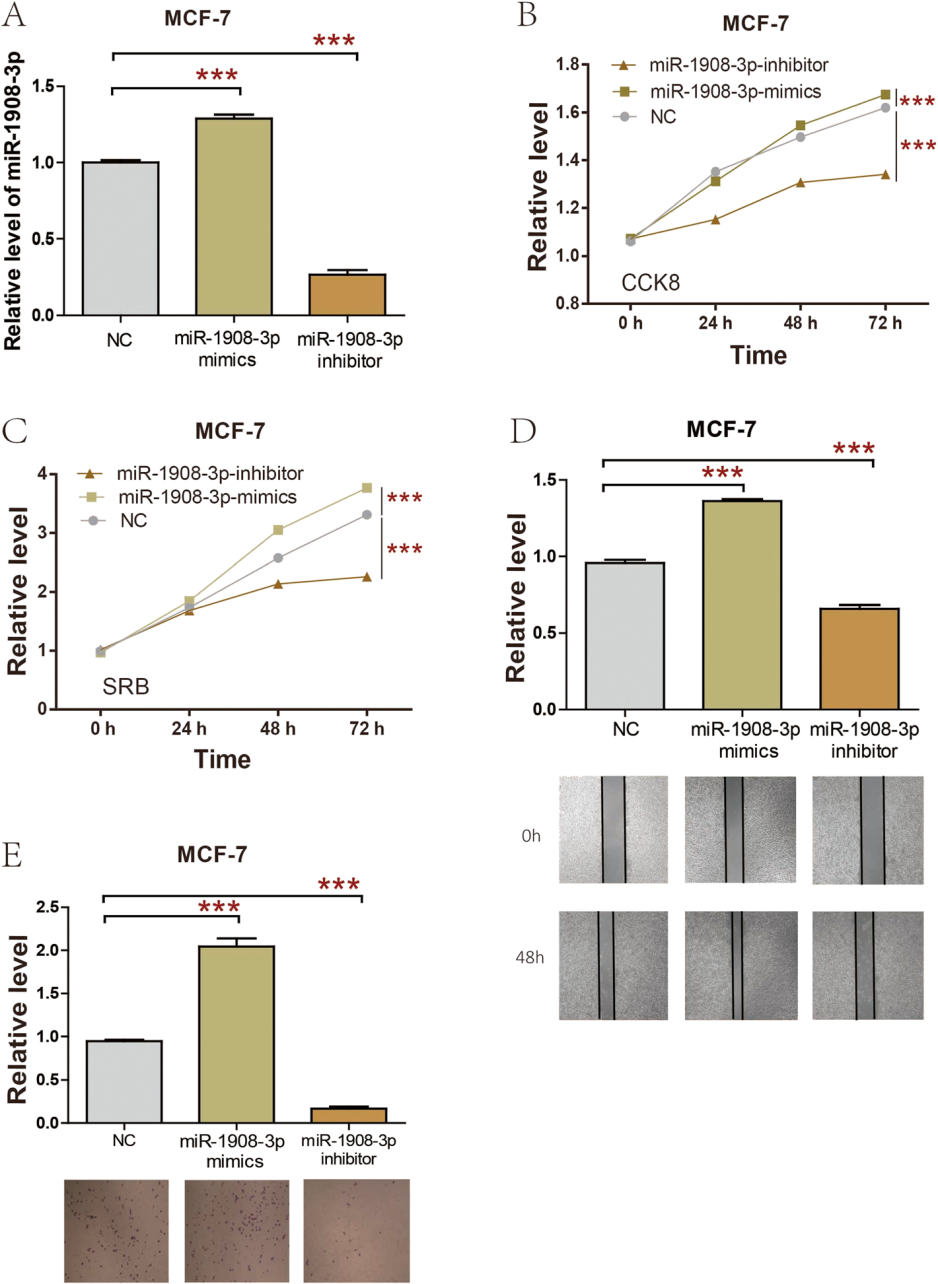
miR-1908-3p promotes MCF-7 cell proliferation, migration and invasion. **a** The expression of miR-1908-3p in MCF-7 cell were affected by transfection of miR-1908-3p mimics or inhibitor. **b** & **c** CCK8 and SRB assay were used to evaluated the proliferation of MCF-7 cells following transfection with miR-1908-3p mimics or inhibitor. **d** The migration ability of MCF-7 cells with miR-1908-3p mimics or inhibitor transfection. **e** The invasion ability of MCF-7 cells with miR-1908-3p mimics or inhibitor transfection. Relative quantification of miR-1908-3p expression were calculated with the 2-∆∆Ct method. ***, *p* < 0.001

In order to study the biological function of miR-1908-3p in breast cancer cells, miR-1908-3p mimics, miR-1908-3p inhibitors and miR-1908-3p negative control (miR-1908-3p-NC) were separately transfected into MCF-7 cells. As presented in Fig. 3a, transfection of miR-1908-3p mimic increased miR-1908-3p level, whereas miR-1908-3p inhibitors significantly inhibited miR-1908-3p level in MCF-7 cells. CCK8 and SRB were applied to explore the function of miR-1908-3p on breast cancer cell proliferation. The results showed that upregulation of miR-1908-3p increased MCF-7 cells proliferation, while down-regulation of the miR-1908-3p level attenuated MCF-7 cells proliferation (Fig. 3b & c). MCF-7 cells migration and invasion were determined by wound healing and transwell assays. As presented in Fig. 3d, miR-1908-3p mimics promoted the MCF-7 cells migration, while MCF-7 cells migration was suppressed by miR-1908-3p inhibitors. Meanwhile, miR-1908-3p mimics promoted MCF-7 cells invasion, while MCF-7 cells invasion was suppressed by miR-1908-3p inhibitors (Fig. 3e).

### miR-1908-3p promoted the proliferation, migration, and invasion of breast cancer MDA-MB-231 cells

**Fig. 4:**
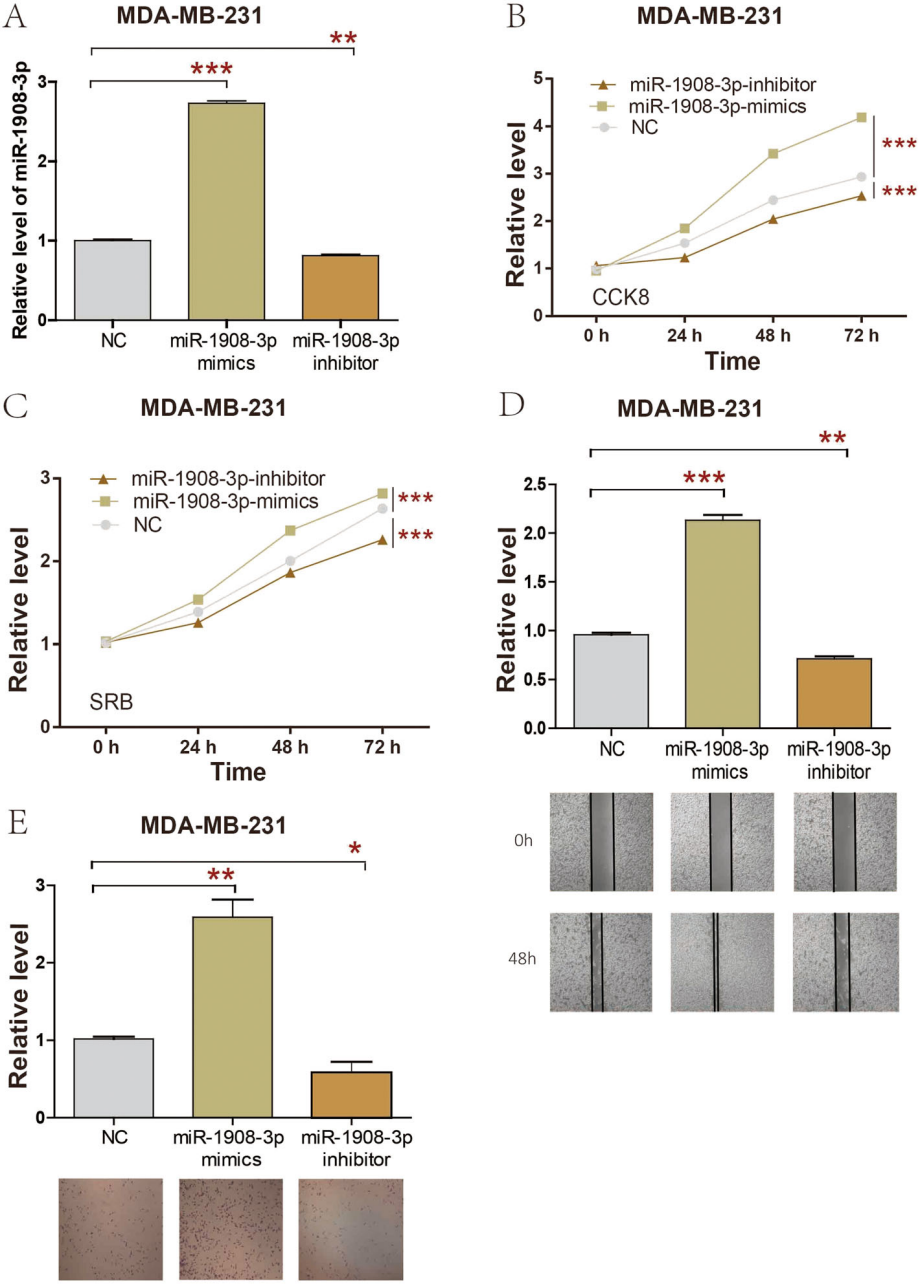
miR-1908-3p promotes MDA-MB-231 cell proliferation, migration and invasion. **a** The expression of miR-1908-3p in MDA-MB-231 cell were affected by transfection of miR-1908-3p mimics or inhibitor. **b** & **c** CCK8 and SRB assay were used to evaluated the proliferation of MDA-MB-231 cells following transfection with miR-1908-3p mimics or inhibitor. **d** The migration ability of MDA-MB-231 cells with miR-1908-3p mimics or inhibitor transfection. **e** The invasion ability of MDA-MB-231 cells with miR-1908-3p mimics or inhibitor transfection. Relative quantification of miR-1908-3p expression were calculated with the 2-∆∆Ct method. *, *p* < 0.05; **, *p* < 0.01; ***, *p* < 0.001

At the same time, miR-1908-3p mimics, miR-1908-3p inhibitors and miR-1908-3p negative control (miR-1908-3p-NC) were also separately transfected into MDA-MB-231 cells. As presented in Fig. 4a, transfection of miR-1908-3p mimic increased miR-1908-3p level, whereas miR-1908-3p inhibitors significantly inhibited miR-1908-3p level in MDA-MB-231 cells. The results of CCK8 and SRB showed that upregulation of miR-1908-3p increased MDA-MB-231 cells proliferation, while down-regulation of the miR-1908-3p level attenuated MDA-MB-231 cells proliferation (Fig. 4b & c). MDA-MB-231 cells migration and invasion were determined by wound healing and transwell assays. As presented in Fig. 4d, miR-1908-3p mimics promoted MDA-MB-231 cells migration, while MDA-MB-231 cells migration were suppressed by miR-1908-3p inhibitors. Meanwhile, miR-1908-3p mimics promoted MDA-MB-231 cells invasion, while MDA-MB-231 cells invasion owere suppressed by miR-1908-3p inhibitors (Fig. 4e). Taken together, these results revealed that the increased level of miR-1908-3p promoted breast cancer cells proliferation, migration, and invasion.

### Exploration of miR-1908-3p target genes

**Fig. 5:**
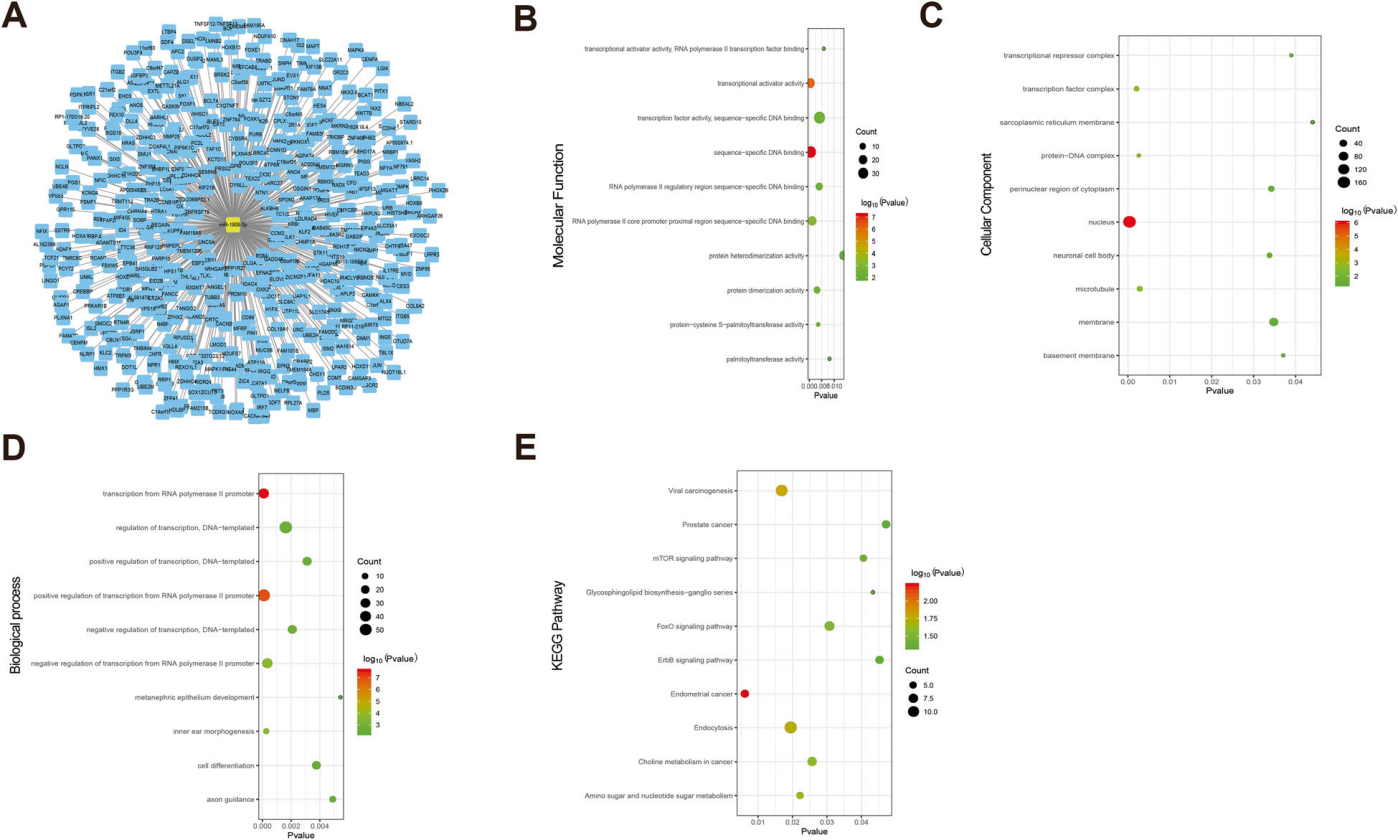
Gene ontology terms and KEGG pathway enriched by the potential target genes of miR-1908-3p. **a** The predicted target genes of miR-1908-3p. **b** The GO Term molecular function enriched by the potential target genes of miR-1908-3p. **c** The GO Term cellular component enriched by the potential target genes of miR-1908-3p. **d** The GO Term biological process enriched by the potential target genes of miR-1908-3p. **e** The KEGG pathways enriched by the potential target genes of miR-1908-3p

To investigate the possible regulation mechanisms of miR-1908-3p, we utilized an online bio-informatics database Targetscan to select possible miR-1908-3p target genes. A total of 480 targeted genes were predicted by Targetscan (Fig. 5a). For better understanding of these genes, GO function and KEGG pathway enrichment analysis were performed. GO functional annotation includes molecular function (MF), cellular component (CC) and biological process (BP). The top 10 enriched GO items were listed in Fig. 5b-d. In the MF of GO analysis, these genes were significantly enriched in sequence-specific DNA binding, transcriptional activator activity, RNA polymerase II core promoter proximal region sequence-specific binding, RNA polymerase II core promoter proximal region sequence-specific DNA binding, protein dimerization activity and protein-cysteine S-palmitoyltransferase activity (Fig. 5b). For CC analysis, these genes were significantly enriched in nucleus, transcription factor complex, protein-DNA complex, microtubule and neuronal cell body (Fig. 5c). BP analysis demonstrated that these target genes were significantly enriched in transcription from RNA polymerase II promoter, positive regulation of transcription from RNA polymerase II promote, inner ear morphogenesis, negative regulation of transcription from RNA polymerase II promoter and regulation of transcription, DNA-templated (Fig. 5d). The result of KEGG pathway enrichment analysis showed that these genes were mostly enriched in endometrial cancer, viral carcinogenesis, endocytosis, amino sugar and nucleotide sugar metabolism and choline metabolism in cancer (Fig. 5e).

**Fig. 6:**
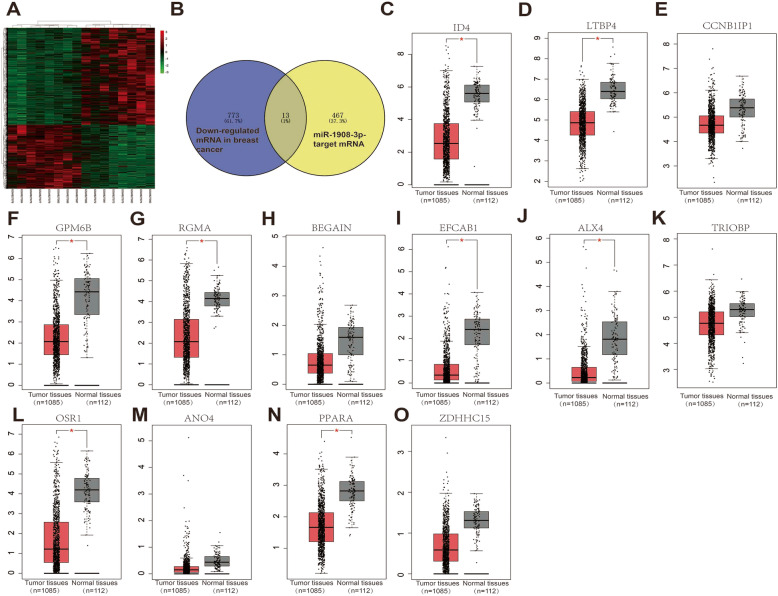
Identification of candidate miR-1908-3p targeted genes. **a** The DEGs between breast cancer samples and normal breast samples from GSE33447 database. **b** The intersection of miR-1908-3p target genes and down-regulated DEGs. **c**-**o** The expression levels of ID4, LTBP4, CCNB1IP1, GPM6B, RGMA, BEGAIN, EFCAB1, ALX4, TRIOBP, OSR1, ANO4, PPARA and ZDHHC15 obtained from the GEPIA database. *, *p* < 0.05

**Fig. 7:**
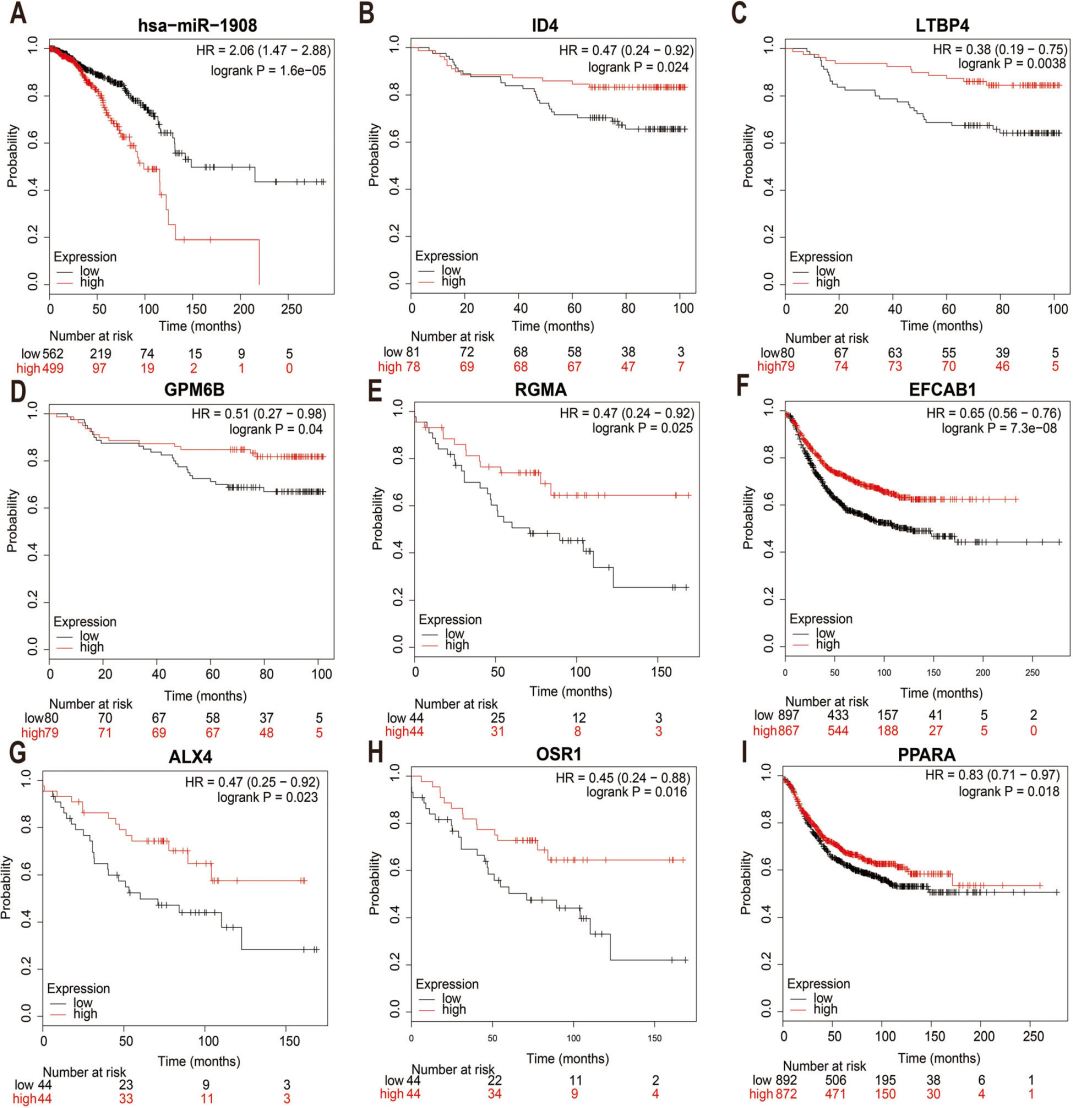
Prognostic analysis of miR-1908-3p target genes. **a**-**i** The prognostic analysis of hsa-miR-1908, ID4, LTBP4, GPM6B, RGMA, EFCAB1, ALX4, OSR1 and PPARA obtained from the Kaplan-Meier Plotter website

It is well known that much evidence supports the negative correlation between expression of miRNAs and target genes. We first identified DEGs (different expression genes) between breast cancer samples and normal breast samples using GSE33447 database (Fig. 6a). Subsequently, 1192 up-regulated mRNAs and 786 down-regulated mRNAs were identified. After conducting a combined analysis of down-regulated mRNAs and target genes of miR-1908-3p, we further identified 13 miR-1908-3p target genes with down-regulated mRNAs in breast cancer samples. These genes were ID4 (inhibitor of DNA binding 4), LTBP4 (latent transforming growth factor beta binding protein 4), CCNB1IP1 (cyclin B1 interacting protein 1), GPM6B (glycoprotein M6B), RGMA (repulsive guidance molecule family member a), BEGAIN (brain-enriched guanylate kinase-associated), EFCAB1 (EF-hand calcium binding domain 1), ALX4 (ALX homeobox 4), TRIOBP (TRIO and F-actin binding protein), OSR1 (odd-skipped related transciption factor), ANO4 (anoctamin 4), PPARA (peroxisome proliferator-activated) and ZDHHC15 (zinc finger, DHHC-type containing 15). Subsequently, GEPIA database was used to detect the expression levels of these 13 genes in breast cancer. As shown in Fig. 6c-o, the levels of eight of the 13 genes were significantly lower in breast cancer tissues than those in normal breast tissues. The expression analysis of CCNB1IP1, BEGAIN, TRIOBP, ANO4 and ZDHHC15 demonstrated no significant difference between breast cancer and normal breast samples. At the same time, the up-regulated expression of ID4, LTBP4, GPM6B, RGMA, EFCAB1, ALX4, OSR1 and PPARA in breast cancer tissues were also observed in GSE33447 (Additional file 2). The prognostic roles of these 8 genes and miR-1908 in breast cancer were evaluated using Kaplan-Meier Plotter website. As shown in Fig. 7a-i, the higher expression of miR-1908 indicated a worse prognosis whereas the higher expression of ID4, LTBP4, GPM6B, RGMA, EFCAB1, ALX4, OSR1 and PPARA correlated with a better prognosis in breast cancer. Based on these findings, a potential miR-1908-3p-mRNA regulatory network, miR-1908-3P-ID4/ LTBP4/ GPM6B/ RGMA/ EFCAB1/ ALX4/ OSR1/ PPARA, contributing to breast cancer onset and progression could be established.

## Discussion

Previous studies have revealed that miR-1908 is abnormally expressed in some malignancies, including glioma, osteosarcoma and liver cancer. The level of miR-1908 was increased and correlated with poor prognosis of glioma patients. The increased level of miR-1908 is not only strongly associated with cell proliferation and migration, but also poor prognosis of osteosarcoma patients. In contrast with its role as an oncogene in glioma and osteosarcoma, miR-1908 may act as a tumor suppressor in liver cancer by targeting MARK1 (Microtubule affinity-regulating kinase 1) signaling pathway. In this research, increased level of miR-1908-3p was observed in breast cancer tissues compared with normal breast tissues, suggesting miR-1908-3p might serve as a diagnostic marker of breast cancer. Meanwhile, we found that the serum level of miR-1908-3p was up-regulated in breast cancer patients compared with healthy volunteers. Furthermore, the young breast cancer patients and HER2-positive patients had a higher level of tissues’ miR-1908-3p than elder breast cancer patients and HER2-negative patients, respectively. The young breast cancer patients had a higher level of serum miR-1908-3p than elder breast cancer patients, the serum level of miR-1908-3p exhibited great reliability in discriminating breast cancer in ROC curve analysis. Considering the number of serum samples in the study, further experiments with enlarged sample size were still needed to verify these results.

MiR-1908-3p is over-expressed in some breast cancer cell lines. We demonstrated that miR-1908-3p could promote breast cancer cell proliferation, invasion and migration, which supports its oncogenic function in breast cancer. MiRNAs play their roles by inhibiting the expression of multiple target mRNAs. Base on the known target gene database Targetscan, 480 mRNAs were predicted to be the target mRNAs for miR-1908-3p. The enriched results of KEGG pathways and GO analysis suggested that most potential target genes are significantly related to transcription. The trio of enriched KEGG pathways, the mTOR (mechanistic target of rapamycin) signaling pathway, FoxO (forkhead box O) signaling pathway and ErbB signaling pathway. Due to the complex interactions between miRNAs and their target mRNAs in vivo, one miRNA may target multiple mRNAs and target mRNAs are usually tissue specific. To test the predicting power and validate the potential target genes of miR-1908-3p in breast cancer, the mRNAs level of these 480 mRNAs were further checked by GEO data and TCGA data. Interestingly, eight genes (ID4, LTBP4, GPM6B, RGMA, EFCAB1, ALX4, OSR1 and PPARA) were confirmed to be down-expressed in breast cancer tissues, and associated with the overall survival time of breast cancer patients, as high expression of these genes correlate with an improved prognosis. These eight genes have greater possibility to be real target genes of miR-1908-3p in breast cancer cells. ID4 protein is a helix-loop-helix DNA binding factor that is involved in cell proliferation and differentiation [29]. The level of LTBP4 was decreased in breast cancer [30]. GPM6B is a membrane glycoprotein that is involved in intercellular communication and membrane transport. Previous studies have found that RGMA inhibits the proliferation of oral squamous cell carcinoma (OSCC) cells, and the low expression of RGMA is closely related to the poor prognosis of patients with OSCC [31]. ALX4 expression was found to be decreased in breast cancer. Meanwhile, ALX4 inhibited breast cancer cell proliferation and metastasis [32]. OSR1 is a tumor suppressor that regulates the proliferation and invasion of renal cell carcinoma cells [33]. Low expression of PPARA is related to the proliferation, invasion and migration of hepatocellular carcinoma cells [34].

Previous research has shown that most of these eight genes act as tumor suppressor genes in multiple types of tumor, including breast cancer. Therefore, miR-1908-3p may target these eight genes to faciliate the progress of breast cancer and decrease the survival time of breast cancer patients.

## Conclusions

In summary, the current research suggested that miR-1908-3p might promote the breast cancer cells proliferation and metastasis by suppressing eight genes (ID4, LTBP4, GPM6B, RGMA, EFCAB1, ALX4, OSR1 and PPARA) and the serum level of miR-1908-3p could be used as a diagnostic and predictive biomarker for breast cancer.

## Supplementary information

**Additional file 1.** Primer sequence used in RT-qPCR.

**Additional file 2.** The expression of eight genes in breast cancer. The expression of ID4 (A), LTBP4 (B), GPM6B (C), RGMA (D), EFCAB1 (E), ALX4 (F), OSR1 (G) and PPARA (H) in breast cancer based on GSE33447.

## Data Availability

The datasets used and/or analyzed during the current study are available from the corresponding author on reasonable request.

## Abbreviations

ID4: Inhibitor of DNA binding 4
LTBP4: Latent transforming growth factor beta binding protein 4
CCNB1IP1: Cyclin B1 interacting protein 1
GPM6B: Glycoprotein M6B
RGMA: Repulsive guidance molecule family member a
BEGAIN: Brain-enriched guanylate kinase-associated
EFCAB1: EF-hand calcium binding domain 1
ALX4: ALX homeobox 4
TRIOBP: TRIO and F-actin binding protein
OSR1: Odd-skipped related transciption factor
ANO4: Anoctamin 4
PPARA: Peroxisome proliferator-activated
ZDHHC15: Zinc finger, DHHC-type containing 15
MARK1: Microtubule affinity-regulating kinase 1
mTOR: Mechanistic target of rapamycin
FoxO: Forkhead box O
TGFB: Transforming growth factor beta
OSCC: Oral squamous cell carcinoma
