# Supplementary material for: Evaluation of MiR-1908-3p as a novel serum biomarker for breast cancer and analysis its oncogenic function and target genes
Source: BMC Cancer. 2020 Jul 10;20:644. doi: 10.1186/s12885-020-07125-4 (PMC7350204; doi:10.1186/s12885-020-07125-4)
Supplement: Supplementary file 1 — Additional file 1. Primer sequence used in RT-qPCR. [file 12885_2020_7125_MOESM1_ESM.docx]

**Additional file 1.** Primer sequence used in RT-qPCR

| Primer name | Primer sequence(5’-3’) |
| --- | --- |
| hsa-miR-1908-3p-F | CCGGCCGCCGGCTCCGCCCCG |
| U6-F | CTCGCTTCGGCAGCACA |
| U6-R | AACTCTTCACTAATTTGCTG |
| cel-miR-39-3p-F | TCACCGGGTGTAAATCAGCTTG |
