# Supplementary material for: Evaluation of MiR-1908-3p as a novel serum biomarker for breast cancer and analysis its oncogenic function and target genes
Source: BMC Cancer. 2020 Jul 10;20:644. doi: 10.1186/s12885-020-07125-4 (PMC7350204; doi:10.1186/s12885-020-07125-4)
Supplement: Supplementary file 2 — Additional file 2. The expression of eight genes in breast cancer. The expression of ID4 (A), LTBP4 (B), GPM6B (C), RGMA (D), EFCAB1 (E), ALX4 (F), OSR1 (G) and PPARA (H) in breast cancer based on GSE33447. [file 12885_2020_7125_MOESM2_ESM.docx]

**Additional file 2. The expression of eight genes in breast cancer**

The expression of ID4 (A), LTBP4 (B), GPM6B (C), RGMA (D), EFCAB1 (E), ALX4 (F), OSR1 (G) and PPARA (H) in breast cancer based on GSE33447.
